# Concepts of multi-level dynamical modelling: understanding mechanisms of squamous cell carcinoma development in Fanconi anemia

**DOI:** 10.3389/fgene.2023.1254966

**Published:** 2023-11-02

**Authors:** Eunike Velleuer, Elisa Domínguez-Hüttinger, Alfredo Rodríguez, Leonard A. Harris, Carsten Carlberg

**Affiliations:** ^1^ Department of Cytopathology, Heinrich Heine University, Düsseldorf, Germany; ^2^ Center for Child and Adolescent Health, Helios Klinikum, Krefeld, Germany; ^3^ Departamento Düsseldorf Biología Molecular y Biotecnología, Instituto de Investigaciones Biomédicas, Universidad Nacional Autónoma de México, Ciudad México, Mexico; ^4^ Departamento de Medicina Genómica y Toxicología Ambiental, Instituto de Investigaciones Biomédicas, Universidad Nacional Autónoma de México, Ciudad México, Mexico; ^5^ Instituto Nacional de Pediatría, Ciudad México, Mexico; ^6^ Department of Biomedical Engineering, University of Arkansas, Fayetteville, AR, United States; ^7^ Interdisciplinary Graduate Program in Cell and Molecular Biology, University of Arkansas, Fayetteville, AR, United States; ^8^ Cancer Biology Program, Winthrop P Rockefeller Cancer Institute, University of Arkansas for Medical Sciences, Little Rock, AR, United States; ^9^ Institute of Animal Reproduction and Food Research, Polish Academy of Sciences, Olsztyn, Poland; ^10^ School of Medicine, Institute of Biomedicine, University of Eastern Finland, Kuopio, Finland

**Keywords:** Fanconi anemia, mechanistic modelling, squamous cell carcinoma, hallmarks of cancer, multi-step tumorigenesis, cancer prevention, cancer treatment, clinical trials

## Abstract

Fanconi anemia (FA) is a rare disease (incidence of 1:300,000) primarily based on the inheritance of pathogenic variants in genes of the FA/BRCA (breast cancer) pathway. These variants ultimately reduce the functionality of different proteins involved in the repair of DNA interstrand crosslinks and DNA double-strand breaks. At birth, individuals with FA might present with typical malformations, particularly radial axis and renal malformations, as well as other physical abnormalities like skin pigmentation anomalies. During the first decade of life, FA mostly causes bone marrow failure due to reduced capacity and loss of the hematopoietic stem and progenitor cells. This often makes hematopoietic stem cell transplantation necessary, but this therapy increases the already intrinsic risk of developing squamous cell carcinoma (SCC) in early adult age. Due to the underlying genetic defect in FA, classical chemo-radiation-based treatment protocols cannot be applied. Therefore, detecting and treating the multi-step tumorigenesis process of SCC in an early stage, or even its progenitors, is the best option for prolonging the life of adult FA individuals. However, the small number of FA individuals makes classical evidence-based medicine approaches based on results from randomized clinical trials impossible. As an alternative, we introduce here the concept of multi-level dynamical modelling using large, longitudinally collected genome, proteome- and transcriptome-wide data sets from a small number of FA individuals. This mechanistic modelling approach is based on the “hallmarks of cancer in FA”, which we derive from our unique database of the clinical history of over 750 FA individuals. Multi-omic data from healthy and diseased tissue samples of FA individuals are to be used for training constituent models of a multi-level tumorigenesis model, which will then be used to make experimentally testable predictions. In this way, mechanistic models facilitate not only a descriptive but also a functional understanding of SCC in FA. This approach will provide the basis for detecting signatures of SCCs at early stages and their precursors so they can be efficiently treated or even prevented, leading to a better prognosis and quality of life for the FA individual.

## Introduction

Rare diseases are disorders that affect less than one case in 2000 people, i.e., only a small percentage of the population. However, there are more than 6000 known rare diseases, affecting over 300 million people worldwide (Boycott et al., 2017; The Lancet Diabetes Endocrinology, 2019). In 80% of the cases, the origin of a rare disease is one or multiple disadvantageous inherited variations of the genome (Wang et al., 2021). These are present in all cell types of the affected individual. Nevertheless, most rare diseases, which are not already prenatally lethal, are rather tissue specific. Pediatricians are more likely confronted with rare diseases than healthcare specialists from other disciplines, as those diseases frequently present symptoms early in life. Rare diseases are often referred to as “orphan diseases,” since in comparison to common non-communicable diseases, such as cardiovascular diseases and type 2 diabetes, there is less research and development of therapies for them. Fanconi anemia (FA) belongs to a small group of rare diseases that are investigated more intensively than most others (Velleuer and Carlberg, 2020). This is also the result of significant contributions from patient organizations in the United States, Germany and many other countries (Wu, 2013).

**FIGURE 1:**
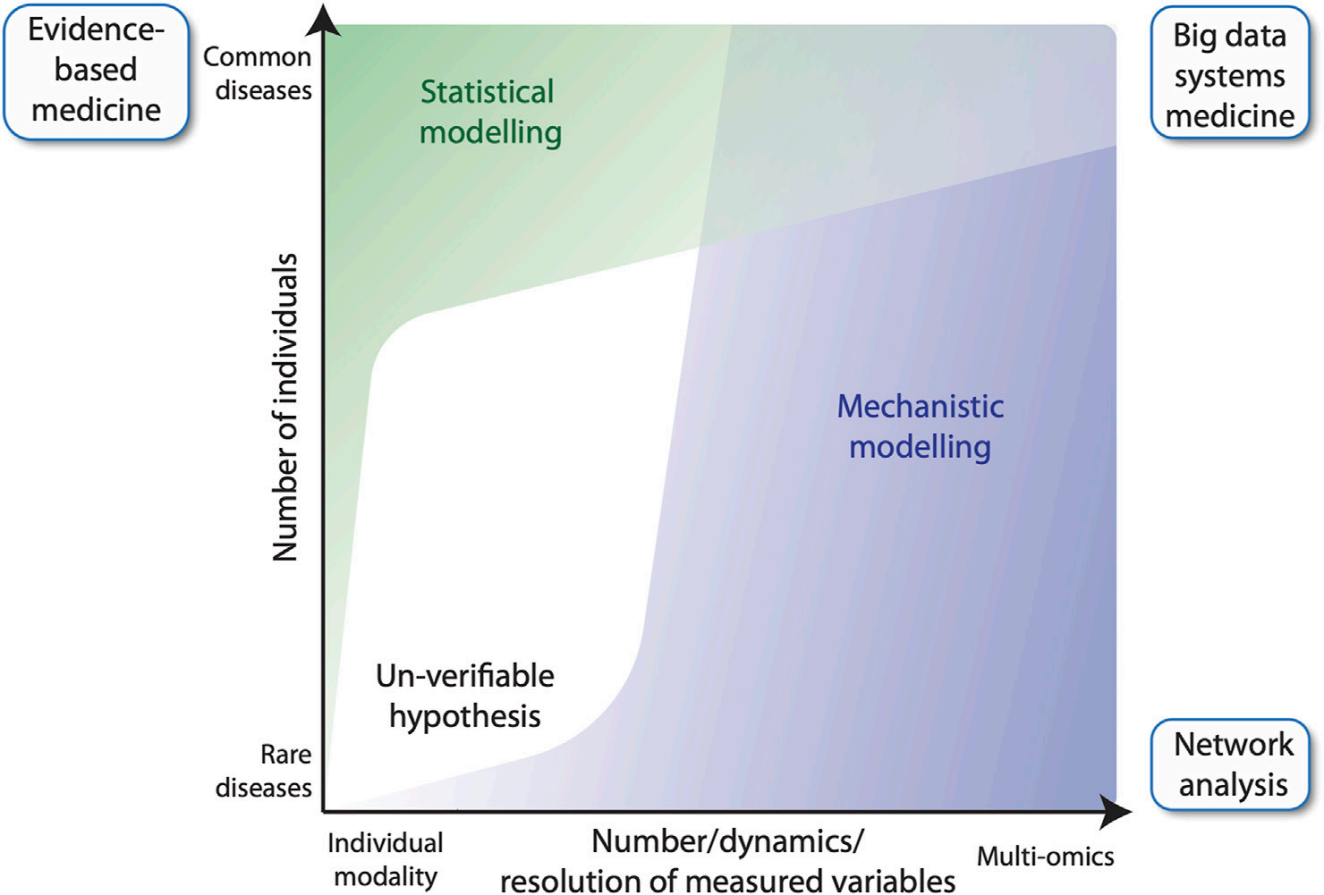
Evidence-based medicine versus network analysis. Number of participants of a clinical study versus the number of variables measured for each individual. In classical evidence-based medicine, many cases and controls are studied for a small number of variables **(top left)**, whereas network analysis, can build upon fewer patients, for which many variables are measured longitudinally **(bottom right)**. The latter benefits from using mechanistic modelling for analyzing data, while evidence-based medicine largely relies on statistical modelling. Big data systems medicine **(top right)** fuses both approaches by integrating large number of data points derived from several patients into predictive mechanistic models.

In general, evidence-based medicine aims to make optimal medical decisions by integrating the experience of a clinician with data from the individual patient and available scientific information on the respective disease (Akobeng, 2005). The latter information often derives from randomized clinical trials involving large numbers of cases and controls. Those trials are the source for the construction of statistical models, i.e., to quantify mathematical relationships between non-random variables measured from the study participants (Figure 1, left). For common diseases, there is no problem identifying a sufficiently large number of cases to achieve acceptable statistical power of the applied statistical model, e.g., reflected by the *p*-value. However, this approach cannot be used for rare diseases due to the small number of cases. An alternative approach is to study a few individuals in very high detail by collecting longitudinal samples for many biological parameters (Wheatley et al., 2021) (Figure 1, right). For example, multi-omic analyses provide many thousands of data points per individual, such as genome-wide DNA methylation, histone modifications and gene expression. These data, together with information on biochemical and regulatory pathways from public databases, such as KEGG (Kyoto Encyclopedia of Genes and Genomes) (Kanehisa et al., 2017), Wikipathways (Pico et al., 2008) and SPOKE (Scalable Precision Medicine Open Knowledge Engine) (Himmelstein and Baranzini, 2015), can then be used to construct multi-level, mechanistic dynamical computational models (Aldridge et al., 2006; Harris et al., 2019; Niarakis and Helikar, 2021). Importantly, mechanistic models differ from statistical models in that they encode existing biological knowledge, often obtained from decades of published experimental studies, into a formal, physics-based mathematical representation that mimics the real living system. Mechanistic models do not require experimental data for their construction, but they do require it to constrain the values of model parameters, which can be numerous. Indeed, the difficulty of estimating values of large numbers of adjustable parameters has been the primary barrier to the widespread utilization of mechanistic models in human health and disease applications. However, the situation is beginning to change. With recent advances in parameter estimation (Eydgahi et al., 2013; Shockley et al., 2018), model analysis and model selection methodologies (Beik et al., 2023), the construction of large-scale, executable models of whole cells, whole tissues and whole patients may now be within reach. Indeed, efforts to develop such detailed computational models, termed “medical digital twins” (Laubenbacher et al., 2021; Masison et al., 2021), are underway. These models can act as virtual platforms for identifying novel therapeutic targets and for designing treatment and preventative protocols to aid clinicians improving individual patient outcomes. In this Perspective article, we introduce the concept of mechanistic modelling as a clinical decision support tool in FA, using the example of the multi-step tumorigenesis of squamous cell carcinoma (SCC) in FA individuals.

## Fanconi anemia: a master example of a rare disease

FA is a rare disease (1 case in 300,000 persons) that was first described nearly 100 years ago by Guido Fanconi (Lobitz and Velleuer, 2006). The clinical characteristics of FA are: i) congenital malformations including absent radius, thumb hypoplasia, disturbed skin pigmentation, as well as inner organ abnormalities most frequently found in the renal and cardiac system (Fiesco-Roa et al., 2019), ii) progressive bone marrow failure already at childhood age (Alter, 2017; Dufour, 2017), and iii) dramatically increased risk of developing cancers, such as acute myeloid leukemia (Alter et al., 2018) and SCC, especially of the head and neck, in early adulthood (Kutler et al., 2003). FA individuals have defects in the molecular machinery of detection and repair of interstrand crosslinks (ICLs) and DNA double-strand breaks (DSBs), which are mostly due to the biallelic inheritance of recessive pathogenic variants in a subset of at least 20 *FANC* genes (*FANCA, FANCC, FANCD1* (*BRACA2*)*, FANCD2, FANCE, FANCF, FANCG, FANCI, FANCJ* (*BRIP1*), *FANCL, FANCM, FANCN* (*PALB2*)*, FANCO* (*RAD51C*), *FANCP* (*SLX4*)*, FANC* (*ERCC4*)*, FANCS* (*BRCA1*)*, FANCT* (*UBE2T*)*, FANCU* (*XRCC2*)*, FANCV* (*MAD2L2*)*, FANCW* (*RFWD3*)) (Wang and Smogorzewska, 2015). Moreover, variants in the *FANCB* gene are inherited in an autosomal recessive X-linked manner, whereas the *FANCR* (*RAD51*) shows an autosomal dominant inheritance pattern and can also be spontaneously mutated (Meetei et al., 2004; Ameziane et al., 2015; Wang and Smogorzewska, 2015). *FANC* genes encode for proteins that maintain genomic integrity during DNA replication, i.e., their inactivation leads to accumulation of DSBs and genomic instability (Ceccaldi et al., 2016). However, patients with identical variants, such as siblings, often show significant differences in their clinical presentation, i.e., there are more factors than the mutated *FANC* genes contributing to the disease (Tischkowitz and Hodgson, 2003; Dufour, 2017).

To date, hematopoietic stem cell transplantation is the only curative treatment option for the hematological complications of FA (Gluckman, 2015) and the main reason for improved life expectancy of young FA individuals (Bonfim et al., 2016). In addition, treatment with supra-pharmacological doses of testosterone analogs, such as Oxymetholone, Danazol and others, can stabilize declining blood counts and even improve them (Scheckenbach et al., 2012; Rose et al., 2014; Paustian et al., 2016; Calado and Cle, 2017). Non-transplanted and, in particular, transplanted FA individuals have a several 100-fold increased risk for developing SCC, especially of their oral mucosa but also in their pharynx, larynx, esophagus, anus, and vulva (Alter et al., 2018), even without the main risk factors like alcohol and tobacco exposure. For adult FA individuals, developing SCC is the most life-threatening complication. Due to their dysregulated DNA repair machinery, FA patients cannot tolerate standard chemo-radiation therapies and treatment side effects are difficult to predict (Lin and Kutler, 2013; Kutler et al., 2016). This makes non-surgical systemic therapeutical options very limited. Therefore, detecting SCC at an early stage and eliminating it surgically is, at present, the best way to prolong the lives of FA individuals (Velleuer et al., 2020). Ultimately, prevention of disease progression should be the goal but is still far away from the clinical routine.

Based on its clinical and cellular phenotype, FA can also serve as a cellular model for the study of general molecular functions and physiological aspects, like aging, as well as other non-communicable diseases occurring in the general population. In that respect, the study of FA has had a major impact on the molecular understanding of breast/ovarian cancer (Howlett et al., 2002). Moreover, *FANC* genes are frequently mutated or dysregulated in sporadic cancers (Del Valle et al., 2020), as well as in childhood cancers (Pouliot et al., 2019). Nevertheless, the enormous and quite specific cancer risk of SCC for FA individuals is poorly understood from a mechanistic standpoint.

## Hallmarks of cancers arising in FA individuals

**FIGURE 2:**
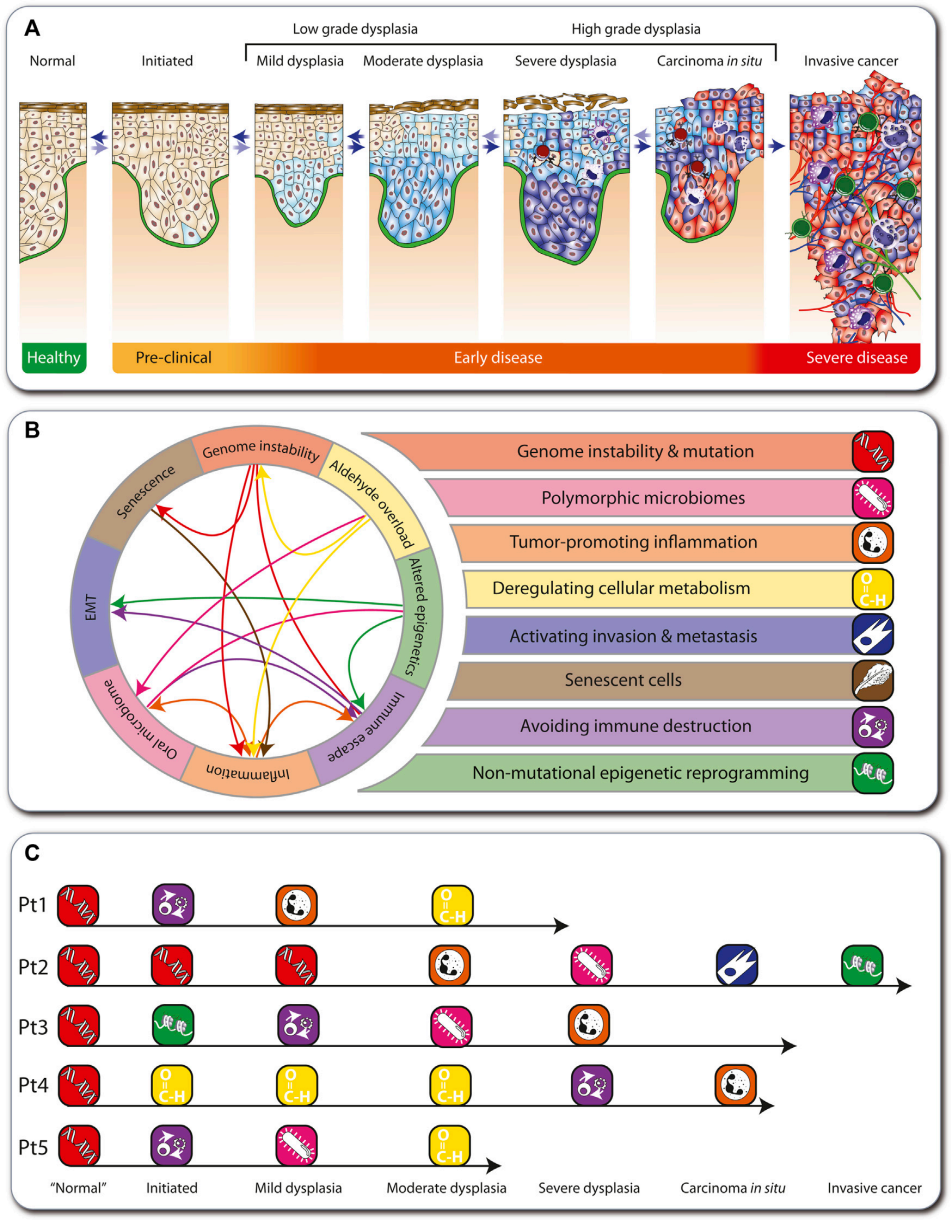
Hallmarks of cancer in FA. Depiction of the multi-step tumorigenesis process **(A)**. Eight major hallmarks describe the onset and progression of cancer in FA individuals **(B)**. In FA, genome instability is the first hallmark to emerge, which influences other hallmarks (arrows). The medical histories of five FA individuals are illustrated as examples such hallmark sequences **(C)**.

During tumorigenesis, the cells of all types of solid cancers arising in adults undergo a multi-step process from a healthy, non-transformed cell to low-grade and high-grade dysplasia, carcinoma *in situ*, and invasive cancer (Carlberg and Velleuer, 2021) (Figure 2A). The early stages of this process are reversible, but when a “point of no return” is reached, an aggressive carcinoma forms, which via the spreading of metastases will eventually lead to patient mortality. In cancer patients in the general population, this multi-step tumorigenesis process takes decades, but in FA individuals it can take only months to a few years.

During tumorigenesis, transformed cells acquire a conserved set of cancer hallmarks, including self-sufficiency in growth signals, insensitivity to antigrowth signals, and tissue invasion and metastasis (Hanahan and Weinberg, 2000). These are altered functional capabilities, the accumulation of which allows malignant cells to survive, proliferate, and disseminate. The hallmarks are outcomes of specific cell fate-controlling regulatory pathways, which in turn are affected by cancer driver gene mutations (Vogelstein et al., 2013). Environmental factors, like lifestyle decisions on diet, physical activity, and smoking, modulate these pathways. In addition, germline mutations may accelerate tumorigenesis like in the Li-Fraumeni syndrome (Carlberg and Velleuer, 2021). In contrast, 100% of all cancers arising in FA individuals (here termed “FA cancers”) are based on heredity of a defective FA/BRCA pathway of DNA repair. Therefore, genome instability and mutation prominently affects other physiological processes, which can result in the emergence of additional hallmarks, such as polymorphic microbiomes, tumor-promoting inflammation, deregulating cellular metabolism, activating invasion and metastasis, senescent cells, avoiding immune destruction, and non-mutational epigenetic reprogramming. These eight hallmarks of FA cancers (Figure 2B) summarize clinical, cellular and molecular observations in the field (Webster et al., 2022) (Box 1). They overlap largely with the latest version of 14 hallmarks of cancer (Hanahan, 2022). However, while in the general population the order in which the cancer hallmarks are established, as well as the underlying mechanisms, varies significantly, in FA cancers the hallmark “genome instability and mutation” always emerges first. For example, we illustrate five real cases of tumorigenesis in FA individuals (Figure 2C; Box 1). Note that these also demonstrate that in FA cancers there is variability in the order of appearance of subsequent hallmarks.

Box 1 **Real world FA patient examples**

**Patient 1:** Diagnosed with FA at age 4 due to bone marrow failure and was transplanted with the bone marrow of his sister. Clinically, no signs for graft versus host disease (GvHD) had been observed but severe viral reactivation complicated the clinical course. He was treated both with anti-viral and immunosuppressive medication. Patient started to drink alcohol at a social occasion at age 18. A visible lesion developed at age 22 at the gingiva and was biopsied revealing moderate dysplasia. Since then, the patient stopped drinking.

**Patient 2:** Diagnosed with FA at age 6. Additionally, he was a carrier of an inherited mutation in the *APC* (APC regulator of WNT signaling pathway) gene. At age 8, the patient needed hematopoietic stem cell transplantation due to bone marrow failure. Unfortunately, he developed severe GvHD. At age 16, he was diagnosed with oral *candida* infection in a lesion at the gingiva. Due to persistence of the lesion, it was biopsied and the diagnosis of SCC was made. After local excision with clear margins, the patient developed 3 months later a local soft tissue metastasis. Further treatment including radiation and CD274 blockage were not able to save the patient and he deceased at age 19.

**Patient 3:** Diagnosed with FA at age 6 due to bone marrow failure. She was treated with anabolic steroids after a period of transfusions and severe infection. The treatment brought the blood counts up but due to the development of clonal hematopoiesis with pre-leukemia, she was transplanted at age 16. At age 21, an oral lesion at the tongue developed and the diagnosis of a *candida* infection was made. However, after initial treatment, the lesion came back showing signs of inflammation. Due to persistence of the lesion, a biopsy was done at age 23 and a severe dysplasia was diagnosed.

**Patient 4:** Started social drinking and smoking at least one pack a day at age of 16. At age 20, a small lesion at the right side of the tongue was noticed by the patient. Clinical diagnosis of a local inflammation was made and the patient was treated over 2 months with immune suppression. Because there was no clinical benefit from the treatment, the patient stopped the medication on his own. Due to growing of the lesion and development of pain, eventually a biopsy was performed, confirming the diagnosis of T1 stage SCC at age 21. Due to the unusual age at presentation, investigations revealed the underlying FA diagnosis. Patient was treated with local excision.

**Patient 5:** Diagnosed with FA at age 8 and directly transplanted. Mild GvHD was clinically present. At age 28, an oral lesion at the gingiva developed. An infection with *candida* was diagnosed but the patient did not get any further treatment. At age 29, the lesion was biopsied due to increase in size. Histologically, a high-grade dysplasia and an invasive *candida* infection were seen.

## Modelling FA cancer development

**FIGURE 3:**
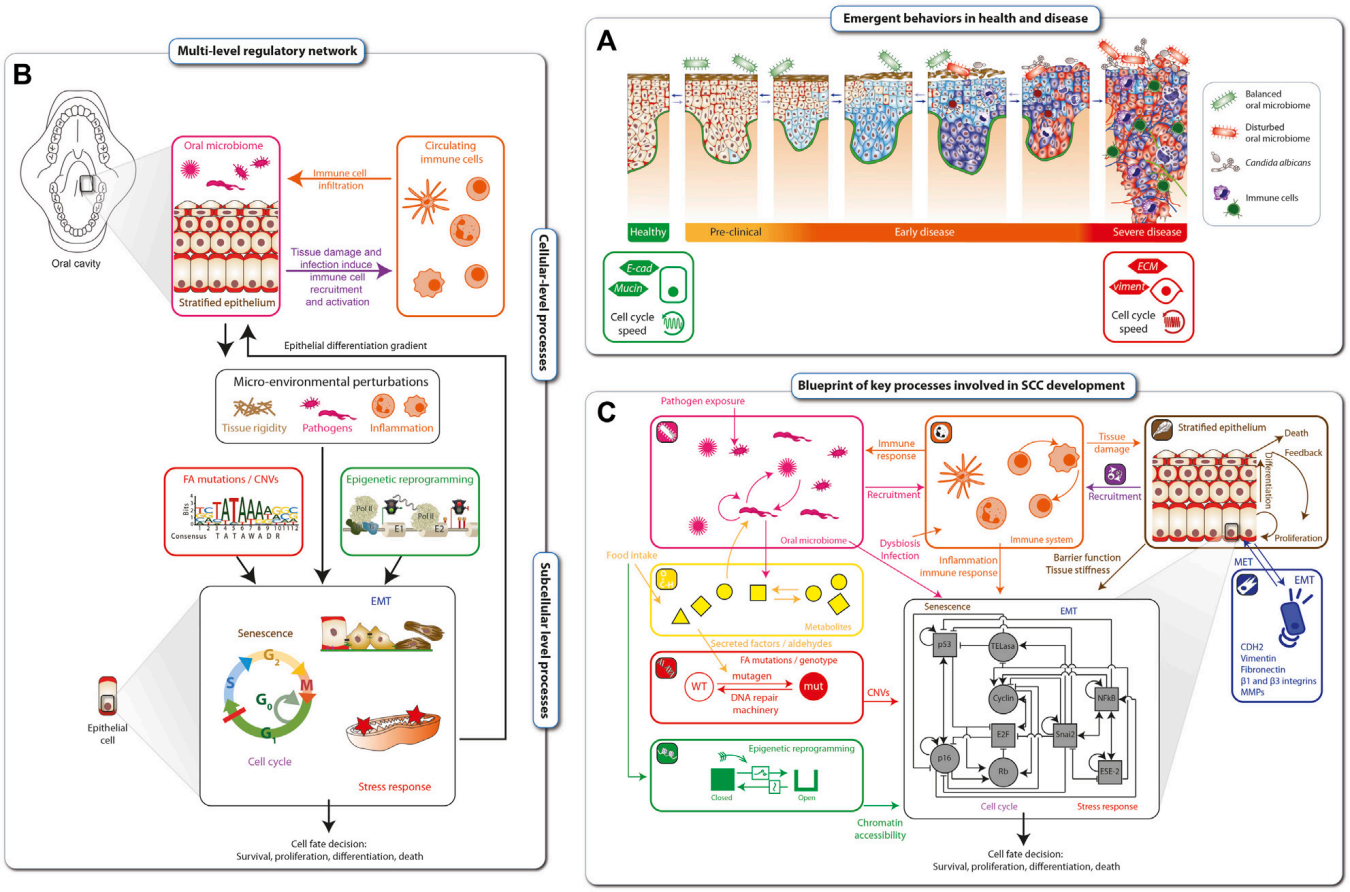
Blueprint for mechanistic modelling of SCC in FA cancer. Disease onset and aggravation emerges as a gradual loss of epithelial tissue function **(A)**. The hallmarks of FA cancer are connected mechanistically through complex multi-level regulatory networks that, under homeostatic conditions, maintain a healthy phenotype **(B)**. The blueprint for modeling SCC development in FA individuals is based on the hallmarks of FA cancer as well as cellular and subcellular regulatory networks. At the cellular level, it includes interplays between the epithelial barrier function, the oral microbiome and the immune responses. Together, these factors shape a microenvironment that is sensed at the sub-cellular level by regulatory networks Driving cell fate decisions. These are targeted by epigenetic and genetic processes including mutations and genomic instability **(C)**.

Cancer progression is a dynamical process ranging from early, mostly clinically asymptomatic, to late stages that are difficult to reverse. Each stage is characterized by a particular configuration (emergent behavior) of the tissue, such as infiltration of immune cells, the microbiome, cell cycle speed and the grade of cell dysplasia (Figure 3A). Disease onset and aggravation emerge from the dynamic interplay between hallmarks of SCC in FA (Figure 2B). The hallmarks are connected mechanistically by complex, multi-level regulatory networks that under homeostatic conditions maintain a healthy phenotype (Figure 3B). At the cellular level, the stratified epithelium underlying the epithelial barrier function interplays with the oral microbiome and the infiltration of immune cells, which together shape the micro-environment of tumor cells. At the sub-cellular level, genetic factors such as mutations in *FANC* genes, copy number variations and epigenetic reprogramming, together with micro-environmental perturbations, such as chronic inflammation and exposure to pathogens or a disturbed microbiome, can lead to altered cell fate decisions. These factors can be intrinsically disturbed, e.g., by aldehydes, but also influenced by lifestyle decisions, such as physical exercise, antibiotic treatment, immunosuppressants or chemotherapy. The complex interplay between these processes makes prevention but also optimal treatment protocols and individual patient assessments extremely challenging without a dynamical computational framework as an aid.

Based on the hallmarks of FA SCC and known underlying cellular and subcellular regulatory networks, we developed a blueprint for modelling SCC development in FA individuals (Figure 3C). Biological processes comprising the blueprint include: i) microbial interactions in the oral cavity (pink inset), ii) circulating immune cells and immune response (orange inset), iii) metabolites affected by food intake or endogenously generated within a cell (yellow inset), iv) DNA damage sensing and repair (red inset), v) epigenetic reprogramming (green inset), vi) stratified epithelial dynamics (brown inset), and vii) loss of epithelial function through EMT (epithelial-mesenchymal transition) (dark blue inset). Together, these tissue level processes are sensed and integrated by the cells and their inner regulatory networks (black inset), resulting in (viii) stress response and xi) cell cycle progression or senescence, x) leading finally to cell fate decisions, including survival, proliferation, cell death/apoptosis. The blueprint amounts to a preliminary wiring diagram connecting these processes and will act as a template for an executable computational model the dynamical multi-level dynamical network underlying the tumorigenesis in FA individuals.

## Principles of mechanistic modelling

**FIGURE 4:**
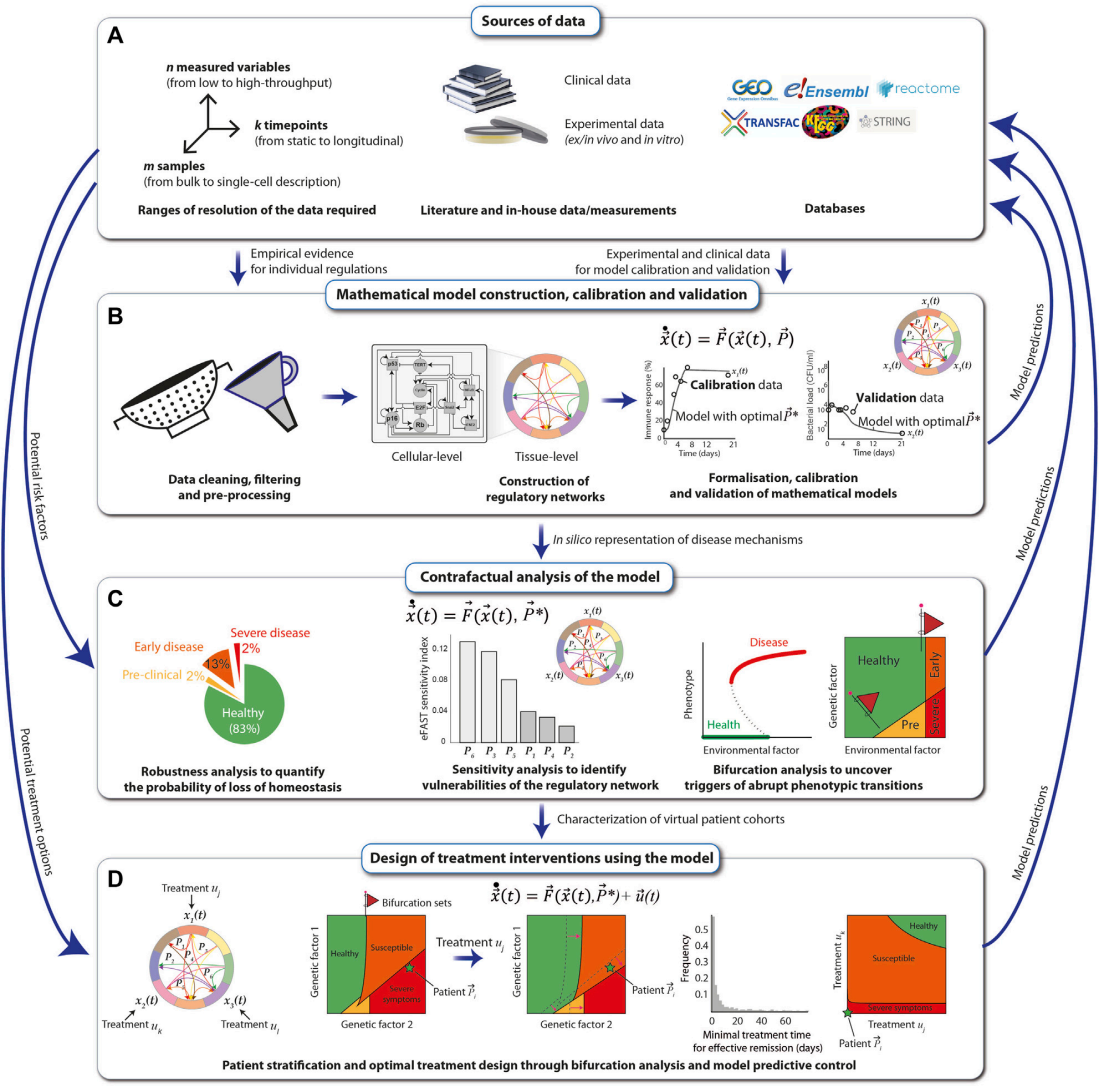
Principles of mechanistic modelling. The preconditions for performing mechanistic modelling of diseases, such as SCC in FA individuals, are clinical data derived directly from patients and experimental data obtained either *in vitro* from patient samples or *in vivo*. Additional data can be obtained from public databases and repositories **(A)**. A mathematical model of regulatory networks is constructed after filtering and processing of the data on the level of either cells or whole tissues. The model is formalized using dynamical systems, which are calibrated and validated with the data **(B)**. The model is analyzed for robustness, sensitivity, and ability to reflect abrupt phenotypic changes in response to perturbations, and resulting model predictions, e.g., the map between a risk factor and the disease severity, are compared with real-world data **(C)**. The mathematical model can be used to systematically explore different types of treatment options for the SCC of an FA individual. Once validated, the most effective predicted treatments may then be applied to patients **(D)**.

The construction, calibration and validation of mechanistic mathematical models require a constant dialogue between the mathematical/computational implementation and analysis of the model, with the experimental and clinical data. As a first step, all available relevant empirical data describing the biological phenomenon are gathered from the literature together with in-house measurements from clinical, animal and *in vitro* studies. These data can be complemented using publicly available databases, such as String-DB (Szklarczyk et al., 2021), KEGG (Kanehisa et al., 2017) and the Human Cell Atlas (Regev et al., 2017). In principle, all types of data, from low-to high-throughput, single-timepoint to dynamic and mean-field to single-patient-single-cell resolution, can be used (Figure 4A).

After cleaning, filtering and pre-processing the data using standard statistical and bioinformatic methods (Sinha, 2014; Sinha, 2015), regulatory networks at the cellular- and tissue-level scales are assembled from the data and visually represented using the Systems Biology Markup Language (SBML) (Keating et al., 2020). The networks are translated into mechanistic mathematical models using a chosen modeling formalism that depends on the scale and resolution of the data. Multi-scale mathematical models can be modularly constructed and assembled using a variety of formalisms from dynamical systems (de Jong, 2002), including Boolean networks for genetic regulatory networks, systems of nonlinear ordinary differential equations (ODEs) for signaling networks and interactions between cell types in the tissue, compartmental differential equations or agent based models to explicitly model spatial coupling between different cell types, stochastic differential equations to simulate population-level distributions of cell markers and delay-differential equations for multi-level regulatory loops that control tissue homeostasis. In general, when constructing a model for a specific disease affecting a particular tissue (e.g., FA-related SCC in the oral mucosa), a good starting point is to identify mathematical models previously proposed and experimentally validated, which can then be extended and adapted to reproduce specific experimental and clinical observations. For example, mathematical models of loss of epithelial homeostasis, originally developed for atopic diseases (Domínguez-Hüttinger et al., 2017), could be extended to include SCC in FA-specific hallmarks. The quantitative models are then calibrated with a training set of experimental data, using global parametric optimization algorithms (Balsa-Canto et al., 2012; Shockley et al., 2018; Tsiantis et al., 2018). This allows one to adapt the mathematical models to specific experimental conditions, as well as to integrate scattered experimental data into a formal and coherent framework that articulates all the individual regulatory players that are typically described in isolation, to understand how they give rise to different clinical manifestations. Finally, model validation is achieved by ensuring that the model can reproduce an additional set of empirical data (the validation set) that was not used for the calibration (Figure 4B).

Next, the calibrated and validated models are analyzed extensively. For this, the nominal conditions, i.e., the calibrated model, are perturbed, e.g., by systematically altering the magnitude of the individual regulatory interactions (changing parameter values), or by structurally altering the different regulatory interactions (changing the equations). The output of the model, e.g., the “phenotype”, is then collected. Examples of such perturbations-to-response mappings are robustness analysis, sensitivity analysis and bifurcation analysis. A robustness analysis tells us which fraction of variations results in a given phenotype (Domínguez-Hüttinger et al., 2017). Sensitivity analysis weights each individual interaction (parameter) in terms of its contribution to the change in the model output (Zi, 2011). In bifurcation analysis, the model output is assessed as one (or more) parameters are gradually changed (Kuznetsov, 2004). Abrupt health-to-disease transitions mathematically correspond to qualitative changes in the model, and they occur typically at bifurcations*.* The value(s) of the parameter(s) at which such a bifurcation occurs, known as a bifurcation (set), is particularly interesting because it represents the critical threshold of a perturbation that can be tolerated by the regulatory structure. Furthermore, the appearance of these bifurcation sets can often be anticipated by early warning signals, such as an increased variance in recovery times of the system (Bargaje et al., 2017). Thus, early warning signals of bifurcating systems can be used to improve early detection strategies for abrupt disease transitions (Figure 4C).

Finally, once the mathematical model has been exhaustively calibrated, validated and analyzed, it can be used for designing and optimising personalised treatment strategies that consider the specific disease stage and patient characteristics (Fey et al., 2015). For this, one starts by identifying the potential targets of the intervention strategy, e.g., growth hormone receptor inhibitors that reduce excessive proliferation of malignant cells, and modifying the model accordingly. Next, analysis of the extended model, i.e., the system without treatments plus the dynamics of the treatments, can be performed (Figure 4C). For example, one can use bifurcation analysis to systematically explore how a given treatment affects the overall virtual population of patients (e.g., considering the natural variations that occur in a population due to polymorphisms) by looking at how the bifurcation sets shift under a specific treatment, such as cetuximab (an epidermal growth factor receptor inhibitor). With a similar line of reasoning, it is possible to use bifurcation analysis to explore how different treatment combinations affect the phenotype of a given patient. Here, again, it is particularly relevant to look for bifurcation sets that separate the qualitatively different clinical phenotypes because these curves correspond to all the minimal treatment combinations that can effectively trigger a disease-to-health transition (Tanaka et al., 2018). Besides bifurcation analysis, other techniques, such as model predictive control (Christodoulides et al., 2017), can also be applied to maximize treatment efficiency while minimizing duration, dosing, and negative side effects (Figure 4D). In all stages of this modelling pipeline, model predictions must be verified by comparing them to empirical data.

**FIGURE 5:**
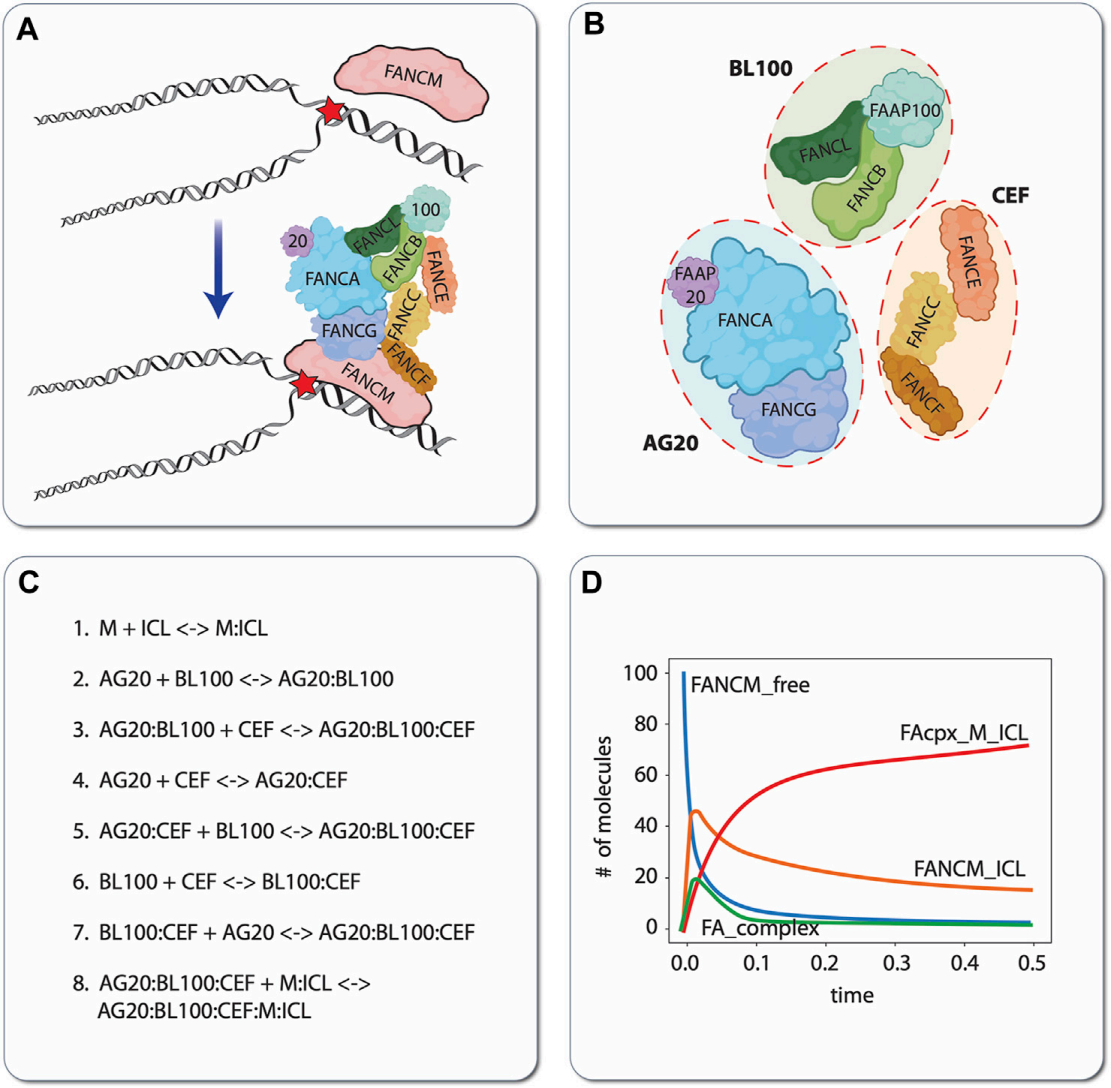
Illustrative example of mechanistic modeling in FA. Schematic of the first two steps of ICL detection and repair, involving binding of the protein FANCM to the DNA, followed by recruitment of the FA core complex **(A)**. For demonstration purposes, we have chosen to model the protein subcomplexes AG20, BL100, and CEF as independent species that reversibly bind to form the FA core complex. Note that this choice of model resolution is at the modeler’s discretion, i.e., alternatively, each protein (FANCA, FANCG, FAAP20, etc.) comprising the subcomplexes could have been modeled as independent species **(B)**. Eight reversible biochemical interactions (16 reactions total) can describe the ICL detection and FA core complex recruitment process **(C)**. *In silico* time courses for different molecular species can be obtained in total by setting values of the binding/unbinding rate constants (all set to 1 in this case) and numerically integrating the resultant set of coupled ODEs **(D)**. ‘FANCM_free’: unbound FANCM; ‘FANCM_ICL’: FANCM bound to the DNA around the ICL; ‘FA_complex’: FA core complex composed of AG20, BL100, and CEF that is not bound to FANCM; ‘FAcpx_M_ICL’: FA core complex bound to FANCM, which is bound to the ICL.

As a simple but illustrative example of the model construction process, we present a small biochemical model of the first few steps of ICL detection and repair by the FA/BRCA pathway (Niraj et al., 2019; Semlow and Walter, 2021). During DNA replication, the presence of an ICL causes the replication fork to stall. This stressed fork is detected by the protein FANCM, which binds to the branched DNA structure caused by replication fork arrest and recruits the FA “core complex” to the damage site (Figure 5A). The FA core complex is composed of three protein subcomplexes, each of which is composed of three proteins (Figure 5B): AG20 is a complex of the proteins FANCA, FANCG, and FAAP20 (FA core complex associated protein 20); BL100 is a complex of FANCB, FANCL, and FAAP100; and CEF is a complex of FANCC, FANCE, and FANCF. For simplicity, we have chosen to represent in the model each of these subcomplexes as distinct molecular species that reversibly bind to each other to form the FA core complex. The complete computational model includes these subcomplex binding reactions, the binding of FANCM to the ICL, and binding of the FA core complex to ICL-bound FANCM (Figure 5C). By defining parameter values for the rates of each of these individual reactions, the model becomes executable, i.e., the populations (or concentrations) of the constituent species can be simulated over time (Figure 5D). In this way, hypotheses regarding the effects of mutations (changing parameter values) and/or adding external perturbagens (e.g., drugs) can be explored *in silico*.

## Experimental data for FA model development and validation: example of multi-omic analysis of a SCC lesion from one FA individual

**FIGURE 6:**
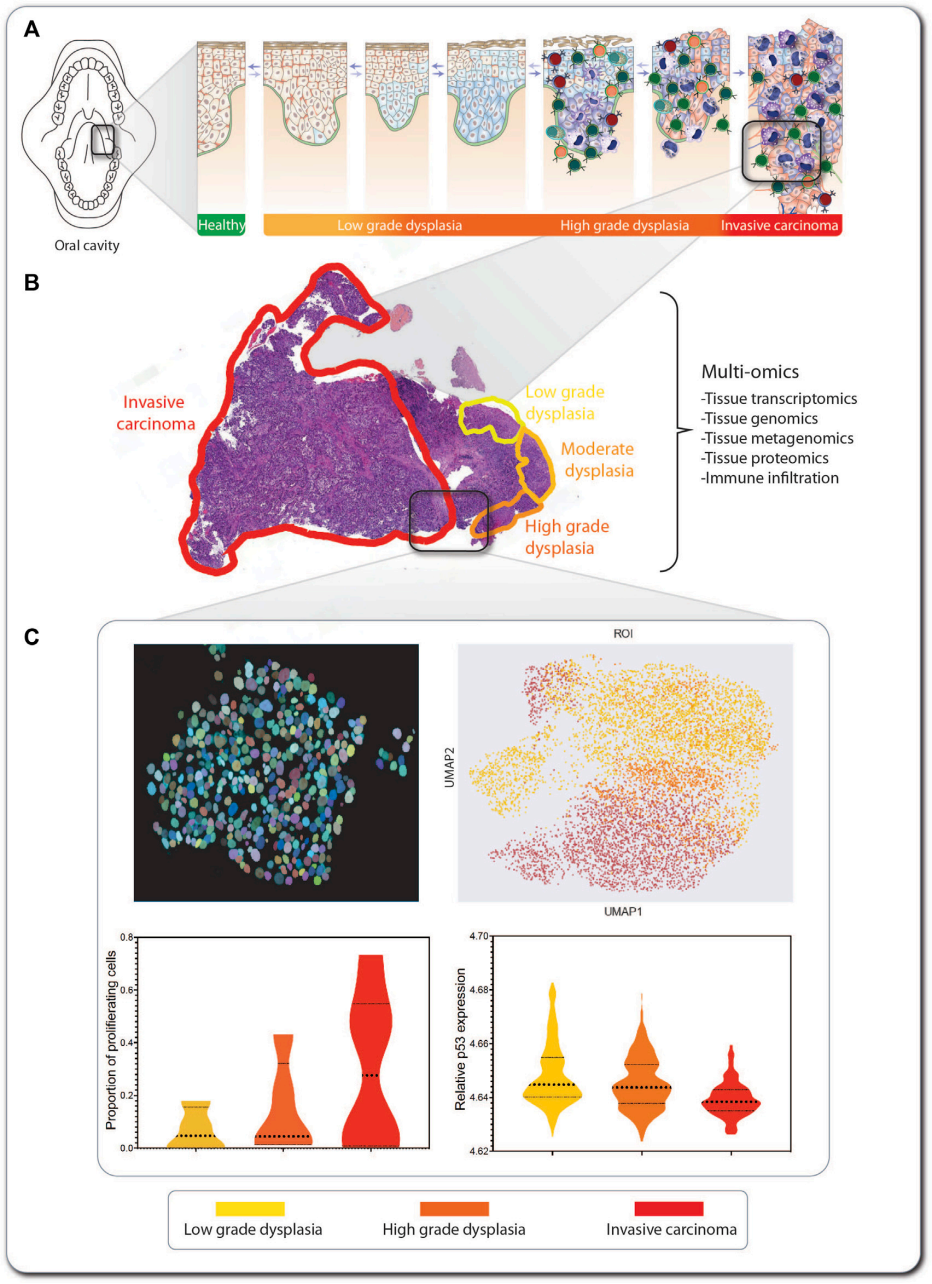
An SCC lesion from a FA patient analyzed by tCycIF imaging. The location within the oral cavity and stage of a hypopharynx tumor from a 41-year-old woman with FA is indicated **(A)**. The hematoxylin- and eosin-stained tumor sample shows multistage carcinogenesis, ranging from low-grade dysplasia (yellow) via high-grade dysplasia (orange) to invasive carcinoma (red) **(B)**. Multi-omic analysis of the tumor includes tissue transcriptomics, genomics, proteomics, and metagenomics for detection of pathogens inhabiting the tumor. Machine learning-based methods are applied in combination with single-cell level segmentation of the tumors and delineation of tumor neighborhoods **(C)**. In this inset of invasive carcinoma, every circle represents an individual tumor cell, and its color indicates its stage within the multi-step tumorigenesis process. The data produced from tumor multi-omics can be processed using non-supervised machine learning algorithms, such as UMAP, for detection of commonalities and divergencies in the tumor sections from multiple patients, and information on markers expression can be extracted from every cell so as to generate graphs for comparing markers expression across the carcinogenic progression.

The tumorigenesis process (Figure 6A) results in a heterogenous composition of tumors, i.e., each tumor contains cells in various stages of the transformation process to aggressively metastasizing cells. Importantly, tumors are not only composed of malignant proliferating cells, but also by multiple cell types, thus making the tumor mass a complex ecosystem that includes immune cells of multiple types (B cells, T cells, macrophages, etc.), tumor-associated fibroblasts, endothelial cells (Anderson and Simon, 2020) and even microbes, including bacteria and fungi (Zong et al., 2023). At the same time, a tumor is not only composed of cells, but also of extracellular matrix and secreted factors that can signal messages among cells (Anderson and Simon, 2020). If malignant tumors from individuals with FA are to be characterized and this information used for accurate model building, all these factors must be accounted for. In this respect, high throughput multi-omics technologies can leverage the components of the tumor of interest, generating data in multiple modalities that need to be integrated and potentially exploited for discovering novel biomarkers and therapeutic targets for individuals with FA.

Of note, classical DNA sequencing, RNA sequencing (RNA-seq), and protein detection technologies are not able to deconvolute and deconstruct the above-mentioned complex composition of a given tumor, since they use the bulk content of the tumor or tissue and are, therefore, constrained to detect the mean expression of molecules, or the presence of a predominant DNA sequence, thus losing information of minor cell populations or incipient emergent cellular clones (Stark et al., 2019). However, we are witnessing the appearance, development, and refinement of multiple technologies with the capacity to resolve the cellular heterogeneity of tumors. Among these technologies, one of the most popular is single-cell RNA-seq (scRNAseq), which has given rise to a growing number of datasets from liquid and solid tumors (after tumor dissociation), as well as healthy tissue, leading to a compendium of single-cell-resolution gene expression atlases of multiple tissues and organs (Yuan et al., 2019). Although scRNAseq is a technology that has revolutionized the resolution at which we analyze cell populations and tissues, it still lacks a critical component, i.e., preservation of tissue architecture in its original context (Stark et al., 2019).

In the context of FA cancer, we are interested in the implementation of technologies that, in a multi-omics fashion, will generate single-cell resolution data but will prevent tissue disaggregation and, therefore, maintain tissue architecture. The latter implies the preservation of cellular neighborhoods and cell-cell interactions, which are lost when the tissue is disaggregated. These technologies are known as spatial omics and include spatial transcriptomics, spatial proteomics, and spatial genomics, which combine molecular characterization with spatial resolution (Akhoundova and Rubin, 2022). The aim of these spatial resolution technologies is to assign omics information to spatial locations in the tissues, reaching cellular and subcellular resolution. Spatial genomics assigns DNA sequencing information, including copy-number variants and somatic mutations; spatial transcriptomics provides information on the number of transcripts of a certain gene per region; and spatial proteomics provides relative amounts of protein concentrations (Akhoundova and Rubin, 2022). The data obtained by these multi-omics technologies are highly dimensional in nature and require potent computational tools for their analysis. Although intense research is underway for improving all spatial omics technologies, the most developed are spatial transcriptomics and spatial proteomics. These technologies will allow for the detection and quantification of cell populations of interest, the discovery of new cell populations, the comparison of the abundance of cell populations across the carcinogenic progression and the quantitative and qualitative description of infiltrating immune cells (Pelka et al., 2021; Karimi et al., 2023). These technologies have the capacity to compensate for the lack of resolution of bulk sequencing analyses, which has hampered the detection of premalignant clones at early stages in bone marrow failure in FA (Rodriguez et al., 2021).

Here, we use as an example a hypopharynx cancer from a 41-year-old woman with FA. The hematoxylin- and eosin-stained tumor sample shows multistage carcinogenesis, ranging from low-grade dysplasia (yellow) to high-grade dysplasia (orange) and invasive carcinoma (red) (Figure 6B). This type of formalin-fixed paraffin-embedded (FFPE) sample can be used for exploration and information retrieval using one or multiple of the multi-omics technologies discussed here (Box 2). If, e.g. tissue-cyclic immunofluorescence (tCycIF) is used, multiple sequential pictures of the tissue stained with fluorescent antibodies will be acquired and stitched. The composite image that is generated must first be segmented using artificial intelligence-based programs, such as ASHLAR (Pelka et al., 2021), which recognize every cell nucleus and apply single-cell-level segmentation of the tumor (Figure 6C, upper left panel). For every cell, we can feature-extract the expression of every marker of interest and proceed to non-supervised machine learning-based algorithms, such as uniform manifold approximation and projection (UMAP) (Figure 6C, upper right panel), which generate clusters of cells based on the similarity of their expressed markers. This allows the separate visualization of cell populations (Becht et al., 2018), such as cancer and immune tumor-infiltrating cells. After feature extraction, we can explore the expression of markers of interest in every tumor population or across the tumor progression, e.g. the proportion of proliferating cells (Figure 6C, lower left panel), the relative expression of p53 (Figure 6C, lower right panel) or other markers of interest.

Box 2 **Multi-omic analysis methods**

Tissue spatial transcriptomics

Tissue spatial transcriptomics allows the characterization of gene expression profiles keeping the tissue’s spatial architecture intact. Multiple techniques have been developed for spatial transcriptomics, mainly based on *in situ* hybridization, *in situ* capturing, *in situ* sequencing or microdissection (Marx, 2021).

Fluorescent *in situ* hybridization (FISH)-based methods exploit the hybridization of fluorescent-labelled RNA-targeting probes with pre-defined transcripts of interest, followed by imaging, visualization, and quantification, which however is limited to the simultaneous detection of a small number of transcripts. Higher efficiency in mRNA detection has been reached with the usage of array-based *in situ* capturing methods. These arrays have attached barcoded oligonucleotides that capture, through complementarity, the mRNAs present in the sample. Capture is followed by reverse transcription to cDNA and NGS, allowing the detection of more than 10,000 targets (Lewis et al., 2021; Maniatis et al., 2021). The widely used Visium technology is next-generation sequencing an example of this approach (Visium spatial gene expression, 10X Genomics) (Stahl et al., 2016).

Recent technologies allow to explore the transcriptome of specific regions of interest in FFPE samples through microdissection. The GeoMx Digital Spatial Profiler by Nanostring allows *in situ* capture of mRNAs using fluorescent-tagged RNA probes, which are linked to UV-photocleavable DNA oligonucleotides of known sequence. The fluorescent-tagged RNA probes are also known as imaging reagents since they will generate a fluorescent image that allows tissue visualization of regions where a specific mRNA is expressed. Once the investigator selects their regions of interest, these areas are exposed to UV light that cleaves the DNA tags in a region-specific manner. This releases indexing oligos that are collected via microcapillary aspiration and dispensed into a microplate and subject to Nanostring mediated counting, or NGS (Merritt et al., 2020). The RNA from FFPE fixed samples very commonly suffers degradation. However, Visium and GeoMx technologies can retrieve good amounts of information from these tissue samples.

Tissue spatial proteomics

The most popular methods of tissue spatial proteomics have the advantage that FFPE samples can be used and, therefore, precious pathological archives can be studied. Strategies for exploring spatial proteomics are based on i) immunofluorescence, ii) imaging mass cytometry by time of flight and iii) sequencing (Lewis et al., 2021). tCycIF is an immunofluoroenscence-based stategy. that uses FFPE tumor and tissue specimens mounted on glass slides that undergo staining cycles. In every cycle, the specimens are stained with fluorochrome-conjugated antibodies and imaged, followed by chemical inactivation of fluorochromes after each round of immunofluorescence (Nirmal et al., 2022). Conventional wide-field, confocal or super-resolution microscopes can be used for image acquisition. After multiple rounds of imaging, a final high-dimensional representation of all the images is assembled into a unique image using computational strategies. The final high-dimension image can be segmented into all individual cells composing the tissue to give single-cell resolution. Neighborhood analysis can also be performed to quantify cell-cell interactions. Of note, tCycIF does not require proprietary reagents, is robust and is a more economical option compared to other spatial proteomics strategies.

CyTOF is a mass spectrometry-based method. In this technology, cellular proteins are detected using antibodies that are conjugated to isotopes from the lanthanide series of rare metals. The sample is imaged using the Hyperion Imaging System^TM^, where these metal-tagged antibodies are laser ablated from regions of interest in the tissue and each ionized metal tag is detected based on differences in their mass instead of the wavelength emitted by a fluorochrome. This technology eliminates the autofluorescence inherent to biological specimens, since the rare metal tags with which the antibodies are conjugated are not present in cells. Also, compensation or background elimination is not needed, since there is no overlap among the signal produced by the ionized metals. In this technology, FFPE samples can be stained with an entire panel of multiple antibodies in a single scanning round without the need for multiple staining and washing cycles. The image is analyzed using a proprietary software (Giesen et al., 2014).

Finally, GeoMx Digital Spatial Profiler by Nanostring can be adapted for detection of proteins instead of transcripts (described above). In this setting, the FFPE tissues are immuno-stained with UV-photocleavable oligonucleotide-labeled antibodies. The spatial location of proteins is again achieved by exposure of the region of interest to UV light that photocleaves the oligos, followed by retrieval of the oligos and sequencing. This provides an average count of oligonucleotides in every region of interest (Merritt et al., 2020; Hernandez et al., 2022).

Tissue spatial genomics

Technologies for spatial resolution of the genome that can preserve tissue architecture are less well developed. Nonetheless, using spatially resolved DNA sequencing will finally deliver information on the process of clonal evolution of solid tumors and provide a timeframe for when a specific mutation appeared. FFPE samples are especially problematic since DNA is very commonly degraded in these specimens (Tang, 2022).

Slide-DNA-seq is one new technology that works with cryo-sectioned intact tissues. Slide-DNA-seq uses cover slip arrays coated with 10 μm DNA-barcoded polystyrene beads, each containing a unique DNA barcode corresponding to its spatial location in the cover slip. This is meant to provide spatial indexing. Then, a 10-μm-thick fresh-frozen tissue section is placed onto the barcoded bead array, treated with HCl for histones removal and treated with the transposase Tn5 to generate DNA fragments that will be flanked with sequencing Illumina adapters. The barcodes are photocleaved from the beads and the resulting DNA sequencing library is amplified by PCR (Tang, 2022; Zhao et al., 2022).

## Toward “digital twins” of FA individuals

Studies on SCC prevention in FA are limited by the small number of individuals with the condition, who are spread around the world. In addition to regular histopathological diagnosis of oral cancer development and cytology-based screening methods (Velleuer et al., 2020), reliable molecular markers are limited. Moreover, the scarcity of genotype-phenotype associations in FA makes it highly likely that each patient will respond in an individual way to drug treatments and/or lifestyle changes. Since robust predictive *in vitro* and *in vivo* FA models are lacking, drug screening and testing cannot be generalized for all FA individuals. For example, *in vitro* analysis of radiation sensitivity of fibroblasts from FA individuals does not correlate with the clinical response of the same patient to radiotherapy (Marcou et al., 2001; Alter, 2002) and the amount of chromosomal breaks found in lymphocyte cultures does not correlate with the severity of the disease, e.g., bone marrow function of the individuum. These issues motivate the effort to create multi-level, dynamical computational models of FA that can aid clinicians in tailoring therapies to each specific FA patient. Models of this type have been termed “medical digital twins” (Laubenbacher et al., 2021; Masison et al., 2021).

Although a consensus definition of a medical digital twin does not yet exist, the concept of a digital twin is common in engineering disciplines (Tao and Qi, 2019). Often referred to as “industrial digital twins,” these models are computational replicas of complex devices or processes, such as jet engines or wind turbines, that are used to diagnose technical problems and guide interventions. Industrial digital twins are typically composed of multiple, interconnected computational models of the constituent components of an engineered system. Critically, this integrated “template” model of the base processes of the engineered system in question is subsequently tuned, or “calibrated,” to a specific instance of that system, e.g., a particular jet engine, using performance data collected from sensors *in real time*. It is this “twinning” process, involving consistent feedback from real-time data streams, that differentiates a multi-level, computational model of a dynamical system from a true digital twin (An et al., 2022). Construction of digital twins for medical and clinical applications has been receiving increased interest lately (Laubenbacher et al., 2021; Masison et al., 2021). However, biological systems are far more complex than engineered systems, making their practical implementation much more challenging. Nevertheless, there have been successful applications of medical digital twins for the treatment of type 1 diabetes (Kovatchev, 2019) and pediatric cardiac patients (Shang et al., 2019). Furthermore, it is important to note that medical digital twins differ from alternative approaches gaining popularity in biomedical sciences, such as statistical and machine-learning models (Swanson et al., 2023), in that they are based on a mechanistic understanding of the underlying biological system. As such, they are not constrained by the confines of the experimental data on which they are constructed, which in FA is sparse.

The utility of a FA medical digital twin will be to aid clinicians in determining best courses of action for individual patients in both the prevention and treatment of malignant tumors. The template model for an FA medical digital twin will comprise the biological processes mentioned previously, including microbiome interactions, DNA damage sensing and repair, EMT, cell cycle progression, and cell death, among others (Figure 3). Calibrating the template model to individual FA patients will be challenging and require collecting spatially resolved, single-cell resolution multi-omics data, epigenome profiling, and metagenomics of the oral microbiome, from patients at regular intervals, e.g., every 3 months in accordance with clinical care guidelines for FA individuals with potentially premalignant lesions in the epithelial tissue. Additional patient data, such as blood draws, genetic screens, and oral swabs, together with standard data from electronic health records, can also be integrated into the calibration data stream. Once the model is personalized in this way, it will be possible to test, *in silico*, numerous preventive and/or therapeutic options before applying them clinically to FA patients. Furthermore, medical digital twins should be flexible and extensible, able to grow in precision and predictive power as new knowledge is accrued and experimental data sets are generated. In this way, the FA digital twin will develop together with the patient and their clinician, ultimately forecasting, with high accuracy and precision, responses to novel personalized interventions and therapies.

However, the here suggested use of mechanistic modelling has a few limitations. Often it is difficult to estimate the values of large numbers of adjustable parameters, which makes the use of mechanistic models in human health and disease a challenge. In addition, most physicians are not used to apply computationally generated recommendations in the general routine of patient diagnosis and care. Furthermore, potentially identifiable data of human subjects might make it difficult to exchange data across country borders.

## Conclusion

For FA cancers, especially oral SCC, the use of multi-level dynamic mechanistic modelling provides a new perspective on early-stage diagnosis and decision support for the treatment of this rare disease. Such an approach is critical, since classical statistical models, using case studies and controls, cannot be applied due to the dearth of large patient groups. As such, we aim to build accurate computational models of tumorigenesis in a limited but representative number of FA patients. These mechanistic models will utilize pre-existing public knowledge on biochemical and regulatory pathways together with our knowledge of the life and disease course of more than 750 FA individuals, which will be essential for distinguishing the tumorigenesis process of FA cancer from that of regular cancer. In this way, our mechanistic models of FA cancer will take specific characteristics of this rare disease into account. Using longitudinal information about the lifestyles of FA individuals over years, together with multi-omics data at the genomic, transcriptomic, and proteomic levels, will lead to the construction of individual-specific models, or digital twins, that can be used to develop personalized treatment options. This approach has the potential to revolutionize the way FA individuals are treated clinically. Since multi-omic data is often used to build the digital twin, ethical approval needs to be obtained. Although recommendations of physicians in the context of disease prevention may not need ethical approval *per se*, but we strongly suggest institutional review board authorization, in particular when there is the aim to publish the findings.

## Data Availability

The original contributions presented in the study are included in the article/Supplementary material, further inquiries can be directed to the corresponding author.

## Abbreviations

APC, APC regulator of WNT signaling pathway; BRCA, breast cancer; CyTOF, cytometry by time of flight; EMT, epithelial-mesenchymal transition; FA, Fanconi anemia; FAAP, FA core complex associated protein; FANC, FA complementation group; FFPE, formalin-fixed paraffin-embedded; FISH, fluorescent *in situ* hybridization; GvHD, graft versus host disease; ICL, interstrand crosslink; KEGG, Kyoto Encyclopedia of Genes and Genomes; NGS, next-generation sequencing; ODE, ordinary differential equation; SBML, Systems Biology Markup Language; SCC, squamous cell carcinoma; scRNAseq, single-cell RNA sequencing; SPOKE, Scalable Precision Medicine Open Knowledge Engine; tCycIF, tissue cyclic immunofluorescence; UMAP, uniform manifold approximation and projection.
